# GENDERED AGEISM: OLDER WORKERS’ NARRATIVES ABOUT AGE

**DOI:** 10.1093/geroni/igz038.2110

**Published:** 2019-11-08

**Authors:** Sarah A Vickerstaff, Mariska F van der Horst

**Affiliations:** 1 University of Kent, Canterbury, Kent, United Kingdom; 2 Vrije Universiteit Amsterdam, Amsterdam, Noord-Holland, Netherlands

## Abstract

Existing research has highlighted that ageism in the workplace may take gendered forms with women ‘never being the right age’ (Duncan & Loretto, 2004). It is further known that individuals have internalised age stereotypes and self-stereotype when being older themselves, also referred to as stereotype embodiment. In this work place based study, through analysis of older workers talk, we examine the extent to which narratives of age differ by gender and work setting. The data base includes 185 participants in five different work settings and different kinds of jobs: blue collar, white collar, managerial, manufacturing and services sectors. Whilst many of the fears about ‘being old’ at work are common across women and men there are some distinct nuances related to the kind of work that people do and others that we argue are gender based.

